# Laboratory-based and free-living algorithms for energy expenditure estimation in preschool children: A free-living evaluation

**DOI:** 10.1371/journal.pone.0233229

**Published:** 2020-05-20

**Authors:** Matthew N. Ahmadi, Alok Chowdhury, Toby Pavey, Stewart G. Trost

**Affiliations:** 1 Institute of Health and Biomedical Innovation at Queensland Centre for Children’s Health Research, Queensland University of Technology, South Brisbane, Australia; 2 Faculty of Health, School of Exercise and Nutrition Sciences, Queensland University of Technology, Brisbane, Australia; California State University San Marcos, UNITED STATES

## Abstract

Machine learning models to predict energy expenditure (EE) from accelerometer data have traditionally been trained on data from laboratory-based activity trials. However, accuracy is typically attenuated when implemented in free-living scenarios. Currently, no studies involving preschool children have evaluated the accuracy of EE prediction models trained on laboratory (LAB) under free-living conditions.

**Purpose:**

To evaluate the accuracy of LAB EE prediction models in preschool children completing a free-living active play session. Performance was benchmarked against EE prediction models trained on free living (FL) data.

**Methods:**

25 children (mean age = 4.1±1.0 y) completed a 20-minute active play session while wearing a portable indirect calorimeter and ActiGraph GT3X+ accelerometers on their right hip and non-dominant wrist. EE was predicted using LAB models which included Random Forest (RF) and Support Vector Machine (SVM) models for the wrist, and RF and Artificial Neural Network (ANN) models for the hip. Two variations of the LAB models were evaluated; 1) an “off the shelf” model without additional training; 2) models retrained on free-living data, replicating the methodology used in the original calibration study (retrained LAB). Prediction errors were evaluated in a hold-out sample of 10 children.

**Results:**

Root mean square error (RMSE) for the FL and retrained LAB models ranged from 0.63–0.67 kcals/min. In the hold out sample, RMSE’s for the hip LAB (0.62–0.71), retrained LAB (0.58–0.62) and FL models (0.61–0.65) were similar. For the wrist placement, FL SVM had a significantly higher RMSE (0.73 ± 0.29 kcals/min) than the retrained LAB SVM (0.63 ± 0.30 kcals/min) and LAB SVM (0.64 ± 0.18 kcals/min). The LAB (0.64 ± 0.28), retrained LAB (0.64 ± 0.25), and FL (0.62 ± 0.26) RF exhibited comparable accuracy.

**Conclusion:**

Machine learning EE prediction models trained on LAB and FL data had similar accuracy under free-living conditions.

## Introduction

Accelerometer based-motion sensors are the most widely recognized and preferred device to objectively measure physical activity in young children [1, 2]. To date, most applications of accelerometry to predict physical activity intensity and/or energy expenditure (EE) have relied on simple linear regression using proprietary activity counts or other representations of acceleration magnitude as the only independent variable [3–6]. Although this approach provides predictions with moderate-to-strong positive correlations with measured EE [7–10], the accuracy of linear regression methods at the individual level of measurement is typically low, with coefficient of variation statistics ranging between 20% to 99% [9, 11]. Further, intensity related cut-points derived from simple linear regression models or receiver operating characteristic (ROC) curves have significant measurement error. Validation studies involving independent samples of children indicate that cut-point approaches misclassify the true intensity of physical activity 35% to 45% of the time [9, 12].

One approach to accelerometer data reduction that has potential to significantly improve device-based measurement of physical activity and sedentary behaviour is pattern recognition or machine learning [13, 14]. Pattern recognition is a branch of artificial intelligence concerned with classifying or describing observations. Widely used in business, robotics, medicine, and engineering, the goal of pattern recognition is to predict future outcomes based on previous knowledge or recognizable features in the raw data [15–17]. State-of-the-art supervised learning algorithms, such as random forest (RF), support vector machine (SVM), and artificial neural networks (ANN) have been shown to be more accurate than traditional simple linear regression approaches at the group and individual level [18–20]. The EE prediction errors associated with these models are 25% to 50% smaller in magnitude than those obtained with regression-based cut-point methods [12].

To date, few investigators have implemented machine learning approaches to derive accelerometer-based EE prediction models for children under five. Due to developmental differences in movement competence and the energy cost of physical activities [21], accelerometer-based EE prediction models developed for adults and school-aged children are not generalizable to preschool-aged children [22]. Zakeri et al. [23] derived accelerometer EE prediction models for preschool-aged children using cross-sectional times-series (CSTS) and multivariate adaptive regression splines (MARS). When evaluated in a whole room calorimeter during the waking hours, root mean square error (RMSE) ranged from 0.05 to 0.10 kcals/min, with the MARS model providing lower prediction errors than the CSTS model. Both prediction models were subsequently cross-validated in an independent sample of 109 children under free-living conditions using doubly labelled water as a criterion measure [24]. RMSE for the CSTS and MARS models was 105 kcals/day (mean percent error = 4.1%) and 139 kcals/day (mean percent error = 7.5%), respectively. Although the models performed well under free-living conditions, they were trained on proprietary activity counts from the ActiGraph accelerometer which cannot be generalized to other makes and models of accelerometers [25].

EE prediction models trained on features in the raw acceleration signal allows for generalizability across different monitor brands and provides greater flexibility for models to map dependencies and nonlinearities in the data [22, 26, 27]. To date, two studies have developed machine learning models to predict EE from raw acceleration features in preschool-aged children. Chowdhury et al. [19] used features in the raw acceleration signal to train multiple linear regression (MLR), ANN, and SVM EE prediction models. Nineteen children completed 10 activity trials encompassing sedentary to vigorous activities while wearing accelerometers on both wrists and the right hip. The RMSE for the best performing wrist (SVM) and hip (ANN) models was 0.63 and 0.55 kcals/min, respectively. More recently, Steenbock et al. [28] trained and evaluated a number of machine learning EE prediction models in a sample of 41 preschool-aged children. Participants completed nine structured activity trials (five preselected, and four chosen by the child) while wearing accelerometers on both wrists, right and left hip, and right thigh, as well as a portable calorimeter. The investigators evaluated mixed-model linear regression, MLR, RF, and ANN prediction models. RMSE ranged from 0.61 to 0.66 kcals/min, with the RF wrist model exhibiting the smallest prediction error.

Although the aforementioned studies show that machine learning EE prediction models are feasible and provide accurate estimates of EE, it is important to note that the models were trained using accelerometer data from laboratory-based activity trials. Previous studies have demonstrated that machine learning models trained on laboratory-based activity trials do not perform well when deployed in free living environments [29, 30]. Therefore, to progress research on the application of machine learning approaches for accelerometer-based physical activity assessment, it is imperative to evaluate the prediction accuracy of laboratory trained EE prediction models under free-living conditions and benchmark performance against EE prediction models trained on true free-living data. However, to our knowledge, no previous studies have developed and tested machine learning EE prediction models for preschool-aged children trained on free-living data. To address this gap in the research literature, the purpose of the current study was to: 1) develop and test machine learning EE prediction models for preschool-aged children trained on features in free-living accelerometer data; and 2) compare the performance of these models against EE prediction models trained on laboratory-based activity trials.

## Methods

### Participants and setting

Twenty-five children between the ages of 3–5 years (mean = 4.1 ± 1.0 y) completed a 20-minute active free play session at a location chosen by the parent or guardian. Children were recruited through a University email list-serve, local media, and word of mouth. The locations of the active free play session included the family home, community parks, and local green spaces. The research team provided age-appropriate toys and play equipment; however, children were free to engage in any activity they desired. This allowed for natural activity behaviour, transitions, and engagement with peers and the environment. The sample was evenly distributed across the age range (36% 3 y, 28% 4 y, and 36% 5 y) and comprised 5 girls and 20 boys. Prior to participation, parental written consent was obtained. The study was approved by the Queensland University of Technology’s Human Research Ethics Committee (1700000423).

### Study design

Fifteen children were randomly assigned to the training sample to develop and evaluate the free-living EE prediction models. The remaining 10 children served as a hold-out test sample to independently evaluate the prediction accuracy of the new free-living EE prediction models and benchmark their performance against previously published laboratory-based EE prediction models for preschool-aged children.

#### Instrumentation

During each active free play session, participants wore a MetaMax 3B portable indirect calorimetry unit (Cortex Biophysical Gmbh; Leipzig, Germany) and an ActiGraph GT3X+ accelerometer (ActiGraph Corporation; Pensacola, FL, USA) on their right hip and non-dominant wrist. During each session, a member of the research team used a Go-Pro Hero 5 (GoPro, Inc, San Mateo, CA) camera to video record participants for subsequent direct observation coding of activity type.

#### Indirect calorimetry

The Metamax 3B portable calorimeter was fitted using a flexible face mask and chest harness, which was appropriately tightened using adjustable straps. The device and chest harness had a total weight of 1.1 kg, making it feasible for use with young children. Following calibration according to manufacturer specifications, respiratory rate, oxygen consumption (VO_2_) and carbon dioxide production (VCO_2_) were measured breath-by-breath using a Triple-V-Turbine, an electrochemical cell and an infrared analyser, respectively. Breath-by-breath VO_2_ data was averaged over a 10 s window and then smoothed using a 60 s moving average [31, 32]. VO_2_ was converted to units of EE (kcal^.^min^-1^) using the Weir equation [33]. To address any confounding related to differences in resting metabolic rate, energy expenditure was also expressed as Metabolic equivalents (METs). METs were calculated by dividing measured energy expenditure by predicted resting energy expenditure, where resting energy expenditure was estimated from the participant’s sex, height, and body mass using Schofield’s equation for children aged 3 to 10 y [34].

#### Accelerometer

The ActiGraph GT3X+ is a small and lightweight activity monitor that measures acceleration along three orthogonal axes with a dynamic range between +/- 8 g and a sampling frequency between 30–100 Hz. For the current study, the sampling frequency was set to 100 Hz. For the hip location, the accelerometer was positioned on the right mid-axilla line at the level of the iliac crest. For the wrist location, the accelerometer was positioned on the posterior side of the arm, between the radial and ulnar styloid processes.

#### Development and evaluation of free-living EE models

There were three steps involved in developing the free-living (FL) EE prediction models: 1) data pre-processing and feature extraction, 2) feature selection, and 3) model training and testing.

#### Data pre-processing and feature extraction

The tri-axial accelerometer signal was converted into a single-dimension vector magnitude (VM) and segmented into 10s non-overlapping sliding windows. Time and frequency domain features found to have utility in previous studies [35, 36] were then extracted from each window for each axis and VM, and included: mean, standard deviation, coefficient of variation, percentiles (10^th^, 25^th^, 50^th^, 75^th^, 90^th^), skewness, kurtosis, maximum, minimum, peak-to-peak, median crossings, zero crossings, sum, mean absolute deviation, power, lag-1 autocorrelation, log energy, inter-quartile range, variance, active samples, number of activations, mean activation interval duration, activation interval duration variability, cross-axis correlations, dominant frequency (0.25 to 5.0 Hz), and dominant magnitude (0.25 to 5.0 Hz). In addition, mean orientation angles for tilt, roll, and pitch were calculated. The resultant feature dataset was synchronized with the measured EE values by aligning date-time stamps.

#### Feature selection

Minimum Redundancy Maximum Relevance (mRMR) feature selection was used to identify features with high discriminative ability [37]. Minimum redundancy favours features that have low dependency to other features without considering how important they are to the outcome variable, whereas maximum relevance selects features that are the most predictive of the outcome variable. The mRMR selection process is based on a balance between these two algorithms, selecting features that derive high relevance and low redundancy. Feature selection was constrained to the 10, 15, and 20 best features.

#### Model training and testing

To replicate the methodology used in previously published laboratory-based (LAB) EE models for preschool-age children, the supervised learning algorithms used in this study were RF and ANN for the hip; and RF and SVM for the wrist. The models were implemented using the “randomForest” [38], “kernlab” [39], “nnet” [40] and “caret” [41] packages within R Software [42]. During the training process each model was optimized using RMSE as the optimization criterion. The hip and wrist RF models were set to 500 trees and the number of features randomly sampled at each node was optimized at three; the hip ANN used a single hidden layer and was optimized at 11 neurons and a weight decay of 0.1; the wrist SVM was configured with a radial basis kernel function and optimized with a cost parameter and gamma estimation of 6.0 and 0.1, respectively. Predictive accuracy was evaluated using leave-one-subject- out cross-validation (LOSO-CV). The performance metrics were the Root Mean Square Error (RMSE) and the Mean Absolute Percent Error (MAPE). MAPE is calculated as the average absolute percent error for predicted EE minus actual measured EE divided by measured EE; and provides an indication of the magnitude of prediction error relative to observed EE.

#### Comparison with laboratory-based EE models

The accuracy of the newly derived free-living EE prediction models were compared to the LAB Hip RF and Wrist RF EE prediction models developed by Steenbock et al. [28], and the Hip ANN and Wrist SVM EE prediction models developed by Chowdhury et al. [19]. Two variations of the laboratory-based models were evaluated; one in which the models were implemented as an “off the shelf” model without additional training, and one in which the models were retrained on the free-living data, replicating the methodology used in the original calibration study (retrained LAB). The performance metrics were RMSE and MAPE.

### Statistical analysis

Differences in RMSE statistics between the LAB, retrained LAB, and FL models were tested for statistical significance using a one-way repeated measures ANOVA. Statistical significance was set at an alpha level of 0.05. When the ANOVA F-ratio was significant, the Fisher LSD procedure was used to determine the locations of significant pairwise differences. Additionally, Bland-Altman plots were created to examine mean bias and 95% limits of agreement (LOA) for total EE expended during the free-living activity session.

## Results

On average, participants expended 33.7 ± 12.2 kcals during the free play session. Based on the direct observation coding of the video files, participants completed a wide range of activities, engaging in energetic play, walking, and running for an average of 11.7 ± 4.0, 2.8 ± 1.9, and 2.4 ± 1.1 min, respectively. The average duration of seated activities was 4.3 ± 2.3 min.

### LOSO cross-validation

The FL prediction models with the best LOSO-CV performance were tested in the hold-out sample. The best performing Hip RF model had 20 features, while the best performing Hip ANN model had 15 features. The best performing Wrist RF and Wrist SVM models both had 15 features. The features selected for the FL models are reported in Table 1. The final FL models along with sample data and R code for implementation can be found in the following link: https://github.com/MA-QUT/Preschool_EE_Models_PLOS_One.

**Table 1:** Features selected for free-living models.

Feature (axis)	RF		SVM	ANN
	Wrist	Hip	Wrist	Hip
10th percentile (x)		✓		
10th percentile (z)		✓		✓
25th percentile (x)	✓		✓	
25th percentile (z)	✓		✓	
50th percentile (vm)		✓		✓
90th percentile (x)	✓		✓	
activation interval duration variability (z)*		✓		✓
coefficient of variation (x)		✓		✓
coefficient of variation (y)	✓		✓	
coefficient of variation (z)	✓	✓	✓	✓
cross-correlation (xz)				
cross-correlation (yz)		✓		✓
dominant frequency (y)	✓	✓	✓	✓
dominant frequency (vm)	✓		✓	
magnitude of dominant frequency (z)		✓		
maximum (x)	✓		✓	
mean absolute deviation (x)		✓		✓
minimum (vm)	✓	✓	✓	✓
minimum (x)	✓	✓	✓	✓
minimum (y)	✓	✓	✓	✓
number of activations (vm)*		✓		✓
number of activations (x)*		✓		
peak to peak (x)		✓		
power (z)		✓		✓
skewness (y)	✓		✓	
skewness (z)		✓		
standard deviation (vm)	✓		✓	
standard deviation (z)		✓		✓
zero crossings (x)	✓	✓	✓	✓
zero crossings (z)	✓		✓	

*Activation interval duration variability and number of activations features were extracted by using the rectified signal with a 4^th^ order Butterworth filter 5Hz lowpass cut off. [43]

RMSE and MAPE statistics for the FL and retrained LAB models are reported in Table 2. The RMSE for the FL Hip RF and ANN models was 0.63 kcals/min (0.96 METs) and MAPE ranged from 27.1% to 28.1%. The retrained LAB Hip RF and ANN models exhibited comparable performance to the FL models with slightly higher RMSE’s (0.65–0.67 kcals/min, 0.99–1.02 METs) and MAPE statistics (28.4%). The FL Wrist RF and SVM models exhibited RMSE’s of 0.63 (0.96 METs) and 0.64 kcals/min (0.99 METs), respectively; with MAPE statistics between 25.4% - 27.4%. The retrained LAB Wrist RF and SVM models exhibited comparable performance to the FL models with RMSE’s of 0.65–0.66 kcals/min (0.99–1.01 METs) and MAPE statistics ranging from 26.0% to 28.3%.

**Table 2:** Leave one subject out cross-validation results for the free-living models and retrained lab model.

	Prediction Model	RMSE		MAPE
		Kcals/min	METs	%
Hip				
Free-Living	RF	0.63 (0.42)	0.96 (0.59)	28.1 (12.0)
	ANN	0.63 (0.43)	0.96 (0.61)	27.1 (11.1)
Retrained Lab	RF Lab	0.67 (0.41)	1.02 (0.57)	28.3 (12.7)
	ANN Lab	0.65 (0.44)	0.99 (0.62)	28.4 (11.8)
Wrist				
Free-Living	RF	0.63 (0.47)	0.96 (0.67)	27.4 (14.0)
	SVM	0.64 (0.51)	0.99 (0.73)	25.4 (12.2)
Retrained Lab	RF Lab	0.66 (0.47)	1.01 (0.67)	28.3 (15.0)
	SVM Lab	0.65 (0.54)	0.99 (0.77)	26.0 (12.6)

Numbers represent: Mean (SD); RF: Random Forest; ANN: Artificial Neural Network; SVM: Support Vector Machine

### Evaluation in hold-out sample

**Fig 1:**
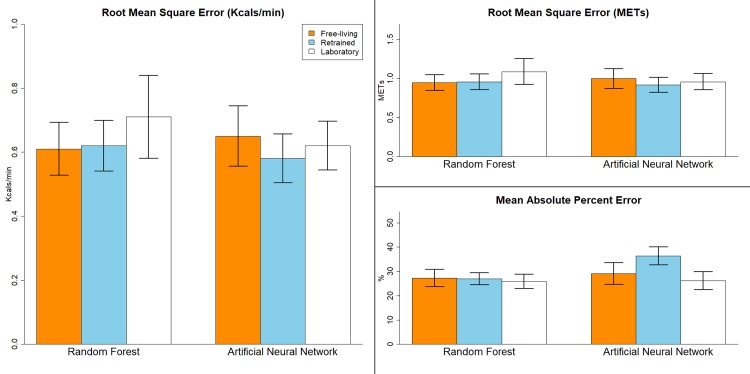
Results for the free-living, retrained laboratory, and off the shelf laboratory models for the hip placement in the hold-out validation sample. Error bars represent standard error.

**Fig 2:**
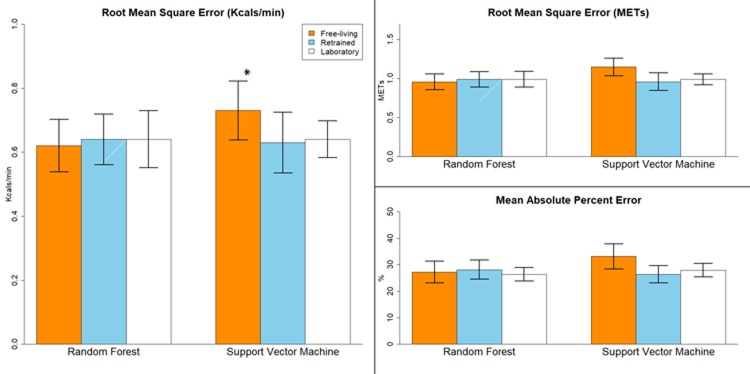
Results for the free-living, retrained laboratory, and off the shelf laboratory models for the wrist placement in the hold-out validation sample. *Significantly different from the retrained Support Vector Machine (p < 0.05) and off the shelf lab Support Vector Machine (p < 0.05). Error bars represent standard error.

RMSE and MAPE statistics for the off the shelf LAB, retrained LAB, and FL models in the hold-out sample are displayed in Figs 1 and 2. For the hip placement (Fig 1), RMSE’s ranged from 0.58 (0.92 METs) for the retrained LAB ANN to 0.71 kcals/min (1.1 METs) for LAB RF. MAPE’s ranged from 25.8% to 36.4%. RMSE’s for the FL and retrained LAB models were similar to their LOSO results and differed by less than 0.06 kcals/min. There were no significant differences in the RMSE’s for the off the shelf LAB, retrained LAB, and FL prediction models (F_5,45_ = 0.85, p = 0.53).

For the wrist placement (Fig 2), RMSE’s ranged from 0.62 for the FL RF model to 0.73 kcals/min (0.96–1.15 METs) for the FL SVM model. MAPE’s ranged from 26.4% to 33.1%. RMSE’s for the FL and retrained LAB were similar to their LOSO results and differed by less than 0.06 kcals/min, with the exception of the FL SVM model which differed by 0.09 kcals/min. RMSE was significantly different across EE prediction models (F_5,45_ = 2.36, p = .05). Post-hoc analysis showed that the RMSE for the FL SVM model (0.73 kcals/min) was significantly higher than the off the shelf LAB SVM (0.64 kcals/min) and retrained LAB SVM models (0.63 kcals/min). No other significant differences were detected.

### Prediction of free play total EE

**Fig 3:**
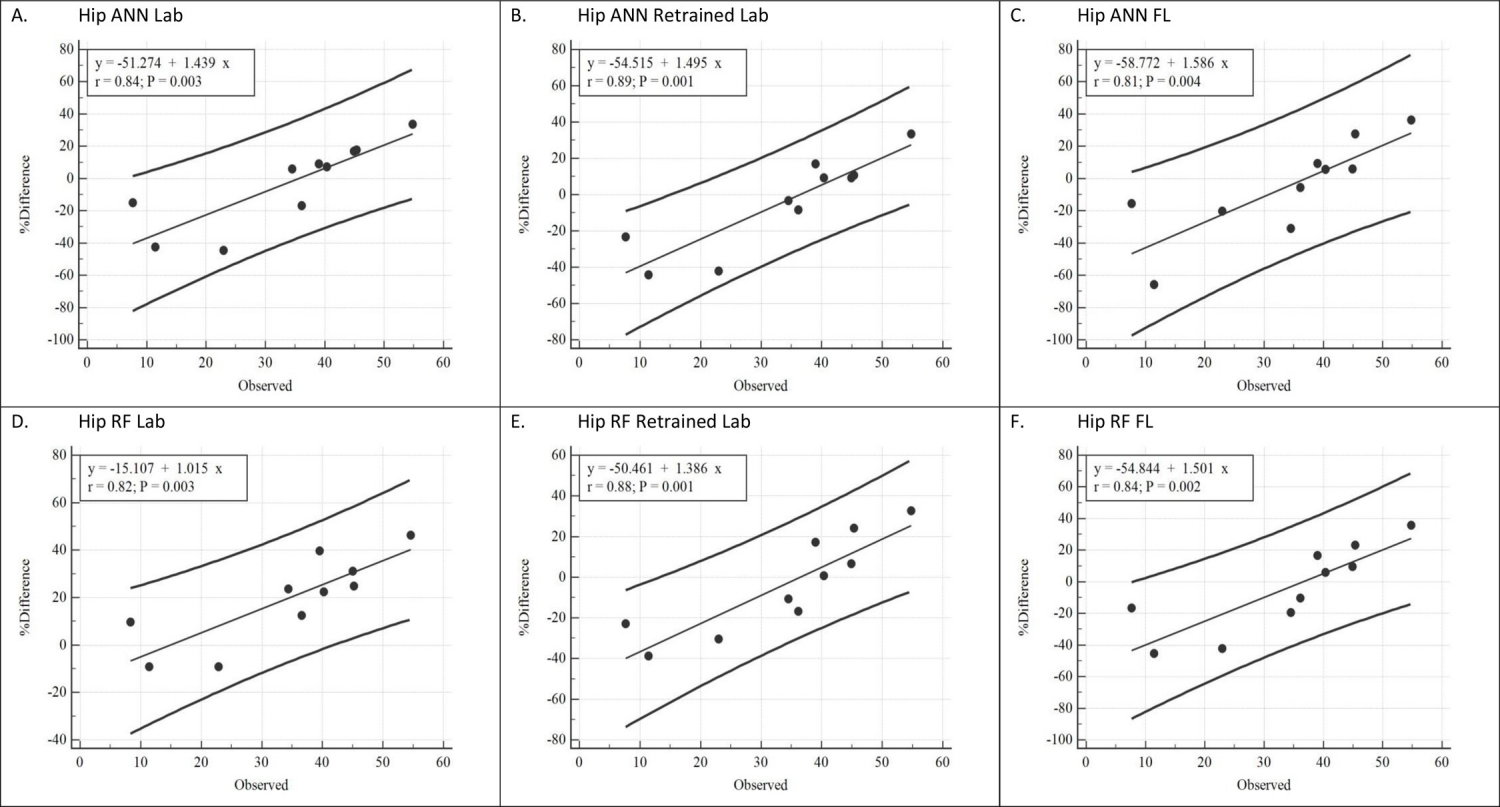
Bland Altman plots depicting regression line and 95% prediction intervals for off the shelf laboratory, retrained laboratory, and free-living models for hip placement. Y-axis values represent percent error (observed–predicted EE). X-axis values represent observed energy expenditure values (kcals).

**Fig 4:**
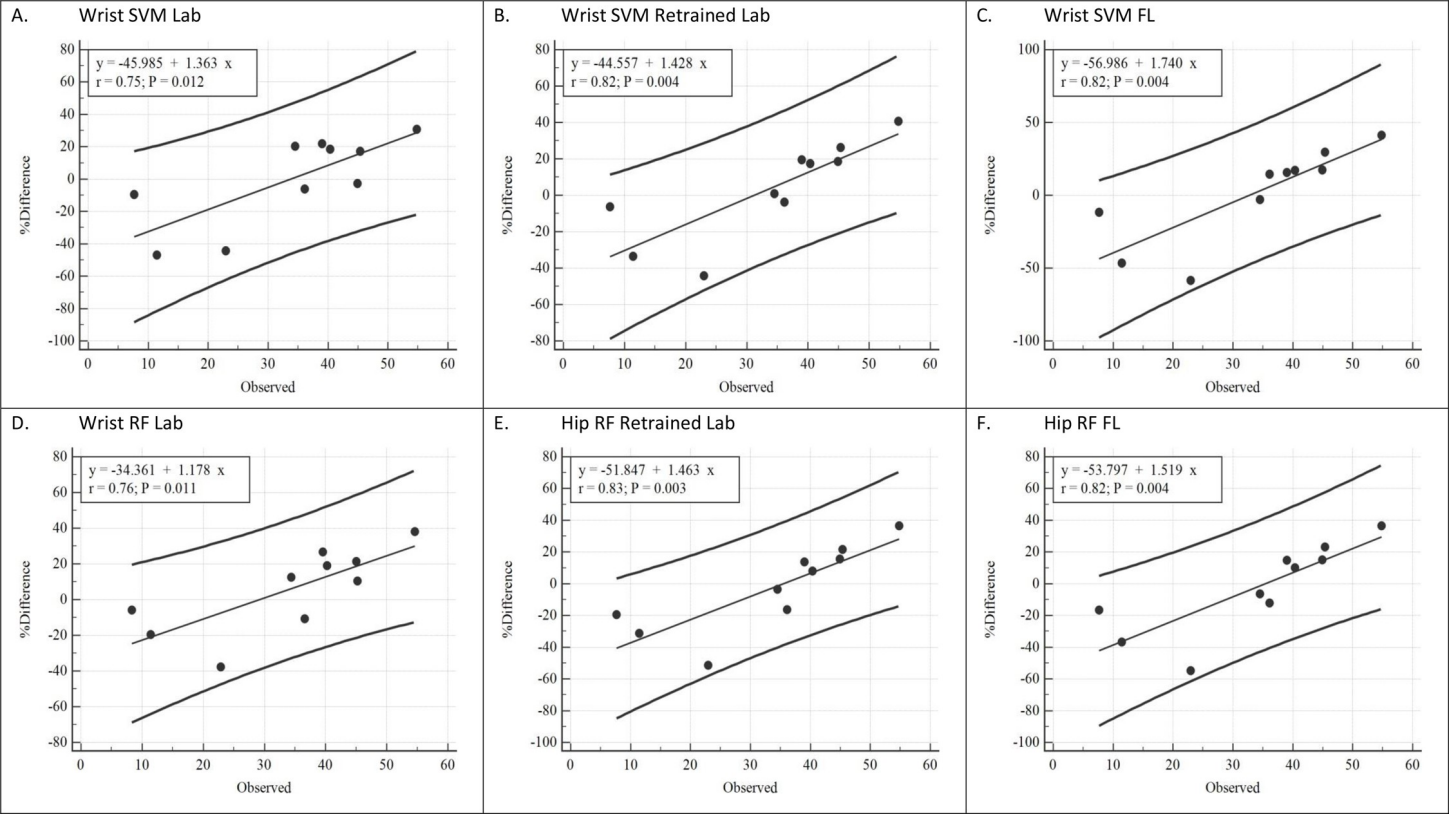
Bland Altman plots depicting regression line and 95% prediction intervals for off the shelf laboratory, retrained laboratory, and free-living models for wrist placement. Y-axis values represent percent error (observed–predicted EE). X-axis values represent observed energy expenditure values (kcals).

Bland-Altman plots depicting the agreement between predicted and observed total EE during the free play session are displayed in Figs 3 and 4. For the hip placement (Fig 3), all six prediction models exhibited evidence of positive proportional bias (r = 0.81 to 0.89) in which EE was overestimated during play sessions with low total EE and underestimated during play sessions with high total EE. With the exception of the LAB Hip RF_,_ the mean bias was not significantly different from zero and predicted EE estimates were within ± 6% of directly measured EE. However, the 95% LOA’s were wide for all models. Based on the line of best fit, the lower and upper prediction limits for an individual ranged from -92.6% to 6.8% at 10 kcals, -55.8% to 33.5% at 30 kcals, and -26.5% to 67.6% at 50 kcals.

For the wrist placement (Fig 4), all six prediction models displayed a similar pattern with evidence of proportional bias (r = .75 to .83). Mean bias was not significantly different from zero and was within ± 6% of directly measured EE. However, for all the models, the 95% LOA’s were wide. Based on the line of best fit, the lower and upper prediction limits for an individual ranged from -92.5% to 21.7% at 10 kcals, -52.3% to 42.8% at 30 kcals, and -26.8% to 80.1% at 50 kcals, respectively.

## Discussion

Machine learning models for predicting EE in young children have been shown to accurate when evaluated under laboratory conditions. However, to advance the application of machine learning methods for the assessment of physical activity, EE prediction models trained on data from laboratory-based activity trials need to be evaluated in independent samples under true free-living conditions. To our knowledge, this is the first study to evaluate the accuracy of machine learning EE prediction models for preschool-aged children under true free-living conditions. Furthermore, this is the first study to benchmark the performance of EE prediction models trained on free-living data against models trained on laboratory-based activity trials. The results indicate that EE prediction models trained on laboratory data exhibit comparable accuracy to models trained on free living data under free-living conditions. The laboratory trained models exhibited RMSE’s within 0.10 kcals/min of the free-living models. Moreover, RMSE’s for the hip and wrist laboratory-trained models were within 0.07 kcals/min and 0.03 kcals/min of their laboratory cross-validation performance, respectively.

Our finding that laboratory-based models exhibit similar prediction accuracy to models trained on free-living data was unexpected and contrary to the results reported in studies evaluating laboratory trained activity classification models under free-living conditions [29, 30]. The discrepancy in findings may be attributable, at least in part, to differences in the inherent variability of the prediction targets. Laboratory trained activity classification models do not generalise well in free living scenarios because children can perform specific physical activities in multiple ways, depending on the child’s motor competence and the constraints imposed by physical and social environment. On the other hand, the resultant energy expenditure of performing physical activities is a physiological response, influenced to a lesser extent by personal and environmental constraints, making the EE predictions of laboratory trained models more robust under free living conditions. In support of this concept, walking style or gait differs so much between individuals it can be used as a biometric identifier, similar to fingerprinting and facial recognition [44]. In comparison, the energy cost of self-paced walking varies much less between individuals, with published MET values for school-aged children ranging from 3.6 to 3.9 METs [45, 46].

With the exception of the LAB Hip RF model, all of the EE prediction models provided acceptable group level estimates of total EE within ± 6% of measured EE. However, the prediction limits for an individual were wide and all models exhibited strong evidence of proportional bias in which EE was overestimated for play sessions with low total EE and underestimated for play sessions with high total EE. The systematically larger prediction errors observed for play sessions with low and high total EE is difficult to explain. However, given the relatively short duration of the free play sessions, and the pulsatile nature of young children’s movement behaviours, there may have been insufficient training instances with low and high physical activity intensity for the models to make accurate predictions across the full physical activity intensity continuum. Notably, the laboratory-based and free-living models were trained on data where 75% of the directly measured EE values were between 1.4 to 2.5 kcals/min (2.2 to 3.8 METs). Therefore, it is possible that the models were less than adequately fitted to provide accurate EE predictions outside this range. As a result, EE for a play session tended to be overestimated if physical activity intensity was predominantly low and underestimated if physical activity intensity was mostly high (see S1 Fig). To increase the accuracy of EE prediction models, future studies should train models using datasets with sufficient number of training instances at the low and high end of the physical activity intensity continuum. This could be achieved through increasing the number of participants and/or the number of active play sessions which would provide opportunities to collect data in a greater variety of environmental contexts that are conducive to low and high physical activity intensity.

The current study had several strengths. Specifically, all models were evaluated in an independent hold out sample of children engaging in unconstrained activities in a variety of locations. Additionally, prediction models for both the hip and wrist were evaluated. The hip and wrist are the most common wear locations for accelerometers [47]. Therefore, the accuracy of models for these two locations have the most relevance for researchers and health practitioners. It is worth noting that, under free living conditions, the wrist and hip models displayed comparable accuracy for estimating EE. Third, the laboratory-based Hip RF model was trained on data collected by a GENEactiv monitor and consistent model accuracy was observed when applied to data collected by an ActiGraph monitor. Despite reported differences in acceleration values recorded by different monitor brands [48, 49], the consistent accuracy of the model between monitors is an indication of generalizability between monitor brands and further supports the use of prediction models trained on raw acceleration data [25, 27].

Offsetting these strengths were several study limitations. First, due to the demands of the data collection protocol, the active play sessions were restricted to 20 minutes. Although participants engaged in a broad range of physical activity behaviours typically performed by preschool-aged children, longer play sessions or multiple play sessions for each participant would have provided the opportunity for more free-living active play evaluations in different environmental contexts which may have allowed for participants to engage in more activities at the low and high end of the physical activity intensity continuum. Second, the study had a relatively small training and hold-out sample. However, the sample size of 25 children provided adequate data to both train and test the machine learning models. With 15 children, the 20-minute free living play sessions generated 1,578,000 data points providing 1,578 10-second windows to train models. With 10 children in the hold-out set, there were 974,000 data points providing 974 10-second windows to test the models. Third, the free-living models did not use lag and lead features from adjacent windows. This would have provided more information for model predictions and/or accounted for the dependence between adjacent windows. However, in order to make direct comparisons with the laboratory-trained models, the free-living models used the same feature sets and did not include information from adjacent windows. Fourth, the study participants were healthy typically developing children. As such, the models evaluated are not generalizable to clinical populations with movement impairments or elevated energy cost of locomotion.

## Conclusions

In summary, when evaluated under true free-living conditions, laboratory-trained accelerometer EE prediction models for preschool-aged children exhibited similar accuracy to models trained on free-living data. Although the laboratory-based models generalized well to a free-living environment and exhibited acceptable accuracy at the group level of measurement, the strong evidence of proportional bias and wide prediction limits exhibited by all the models suggests that they may be inappropriate for predicting EE in individuals. To improve predictive accuracy, future studies should train models using accelerometer data with enough training instances of physical activity with low and high EE for accurate prediction over the complete physical activity intensity continuum. In addition, the inclusion of physiological sensor data such as heart rate or person-level features such as height and weight may improve accuracy.

## Supporting information

S1 Fig Density distribution of observed kcals/min for training data and scatter plot of predicted and observed kcals/min in hold-out data. Orange indicates observed EE between 1.4 and 2.5 kcals/min; Blue indicates observed EE <1.4 and >2.5 kcals/min.
(DOCX)
