# Supplementary material for: Laboratory-based and free-living algorithms for energy expenditure estimation in preschool children: A free-living evaluation
Source: PLoS One. 2020 May 20;15(5):e0233229. doi: 10.1371/journal.pone.0233229 (PMC7239487; doi:10.1371/journal.pone.0233229)

**Prediction of total EE predictions in hold-out validation sample**

The laboratory-based and free-living models were trained on datasets with a positively skewed distribution of EE values with a large proportion of EE values between 1.4 kcals/min to 2.5 kcals/min. As a result, the models were less than adequately fitted to provide accurate EE predictions outside this range. This contributed to the over-prediction of EE values below 1.4 kcals/min and the under-prediction above 2.5 kcals/min. Supplemental Figure 1 depicts the distribution of the EE values used in the training set for the free-living models. The resultant EE predictions from the free-living models compared to ground-truth EE are then presented as scatterplots with a line of identity. The ground-truth EE values between 1.4 kcals/min and 2.5 kcals/min are shown in orange, while EE values outside of this range are shown in blue.


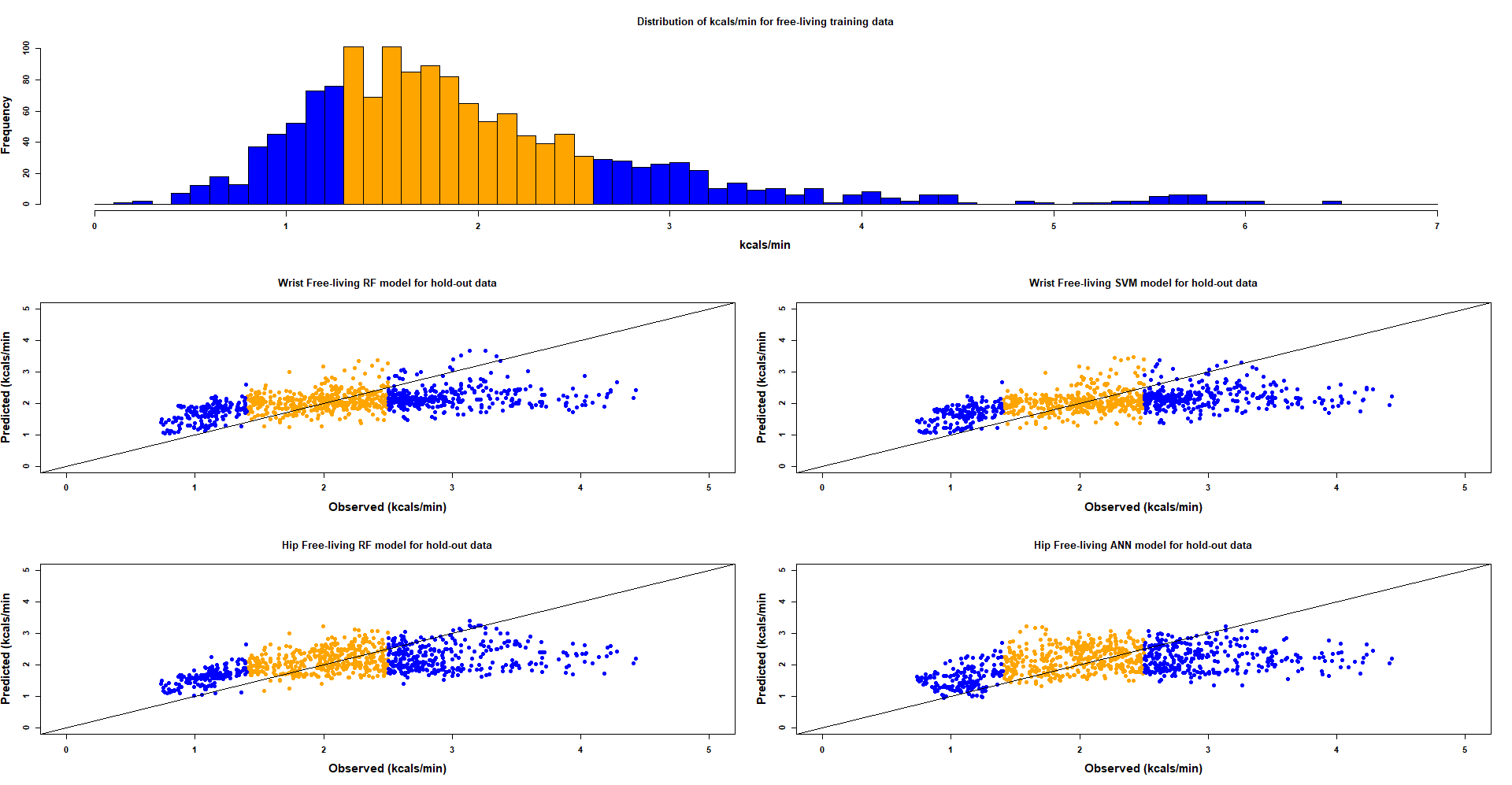

Supplement: S1 Fig — Orange indicates observed EE between 1.4 and 2.5 kcals/min; Blue indicates observed EE <1.4 and >2.5 kcals/min. (DOCX) [file pone.0233229.s001.docx]
